# Gigahertz streaking and compression of low-energy electron pulses

**DOI:** 10.1063/4.0000235

**Published:** 2024-04-01

**Authors:** Dennis Epp, Benjamin Schröder, Marcel Möller, Claus Ropers

**Affiliations:** 1 Department of Ultrafast Dynamics, Max Planck Institute for Multidisciplinary Sciences, 37077 Göttingen, Germany; 2 4th Physical Institute-Solids and Nanostructures, University of Göttingen, 37077 Göttingen, Germany

## Abstract

Although radio frequency (RF) technology is routinely employed for controlling high-energy pulses of electrons, corresponding technology has not been developed at beam energies below several kiloelectronvolts. In this work, we demonstrate transverse and longitudinal phase-space manipulation of low-energy electron pulses using RF fields. A millimeter-sized photoelectron gun is combined with synchronized streaking and compression cavities driven at frequencies of 0.5 and 2.5 GHz, respectively. The phase-controlled acceleration and deceleration of photoelectron pulses is characterized in the energy range of 50– 100 eV. Deflection from a transient space-charge cloud at a metal grid is used to measure a fourfold compression of 80−eV electron pulses, from τ=34 to τ=8 ps pulse duration.

## I. INTRODUCTION

**FIG. 1.**
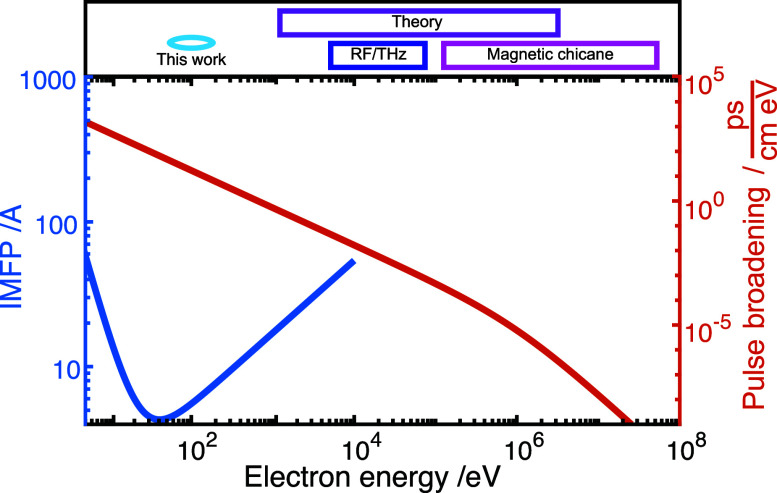
In the top, different compression techniques can be found in the literature.^28,31–37,40–42,44–48,57–72^ There is a noticeable focus on the energy range from 10 to 200 keV. Especially at higher energies, the compression methods change from RF/THz cavities to magnetic techniques. With increasing electron energy, the pulse broadening (red curve) decreases. However, for the study of structural dynamics in monolayers and at surfaces, low-energy electrons are of interest due to the global minimum of the inelastic mean free path (blue curve) for electrons in materials.^73^

Electron beams are powerful probes of material structures and excitations due to their large scattering cross sections and the availability of tunable electron optics for diffraction and microscopy. Pulsed electron beams, typically produced by photoemission, allow for the study of non-equilibrium structural evolution on the intrinsic time and length scales of light-induced dynamics, with examples in the mapping of structural phase transformations,^1–5^ strain dynamics,^6,7^ and lattice equilibration.^8–12^ The temporal resolution of ultrafast electron diffraction (UED) instruments is governed by the electron pulse duration at the location of the sample, which is affected by the initial velocity distribution of the photoelectrons, their mutual Coulomb interaction,^13,14^ and the total propagation time or path length. Today, electron pulse durations in the femtosecond range are routinely achieved at kinetic energies beyond tens of kiloelectronvolts,^15–24^ to a large degree by implementing compact electron sources for a given energy range.^16,25–27^ Moreover, active pulse shaping using various techniques has been implemented, including radio frequency (RF) compression,^28–39^ reflectron compression,^40–43^ and magnetic chicanes.^36,44–49^ Figure 1 (top) displays a set of references from the literature on the respective energy scale. A strong emphasis on experiments at high electron energies is evident. However, the detrimental effects of pulse dispersion are particularly severe at low electron kinetic energies, as illustrated by considering the pulse broadening in picoseconds per cm propagation distance and electronvolt initial energy broadening (Fig. 1, red curve).^23,38,50^

Yet, due to the few-Ångstrom inelastic mean-free path of electrons at kinetic energies of tens of electronvolts (Fig. 1, blue curve), this low-energy regime is highly relevant for structural dynamics studies of surface reconstructions, adsorbates, as well as mono- and bilayers.^51^ Recently, our group developed ultrafast low-energy electron diffraction (ULEED) in transmission^26^ and reflection,^11^ with first applications in the observation of adsorbate dynamics,^52^ phase-ordering kinetics,^11,53^ lattice thermalization,^54^ and the coherent control of structural phase transformations.^55,56^ In order to achieve pulse durations down to 1 ps, miniaturized electron guns^27^ were developed that reduce total propagation distances to the order of a few 100 μm. However, besides a further reduction of the dimensions of electron guns, active pulse control schemes remain highly desirable also in the typical energy range of LEED.

In this work, we demonstrate radio frequency compression of ultrashort low-energy electron pulses. Specifically, we use an RF compression cavity to manipulate the phase-space distribution of 50- to 120-eV electron pulses from a miniaturized photoelectron source. This allows the electron pulse duration to be reduced and the temporal focus position along the propagation path to be adjusted. We use a streak cavity to demonstrate the synchronization between the electron pulses and the RF field and to estimate the uncompressed pulse duration. The longitudinal phase-space distribution is manipulated by a compression cavity and then analyzed by a retarding field method as a function of compression phase. This provides phase-dependent characterizations of the average kinetic energy and the energy width. Finally, time-dependent lateral deflection from a photogenerated space-charge cloud is used to fully characterize the impact of the compression cavity by measuring the pulse duration for three different electron energies.

## II. EXPERIMENTAL SETUP AND ELECTRONIC SYNCHRONIZATION

**FIG. 2.**
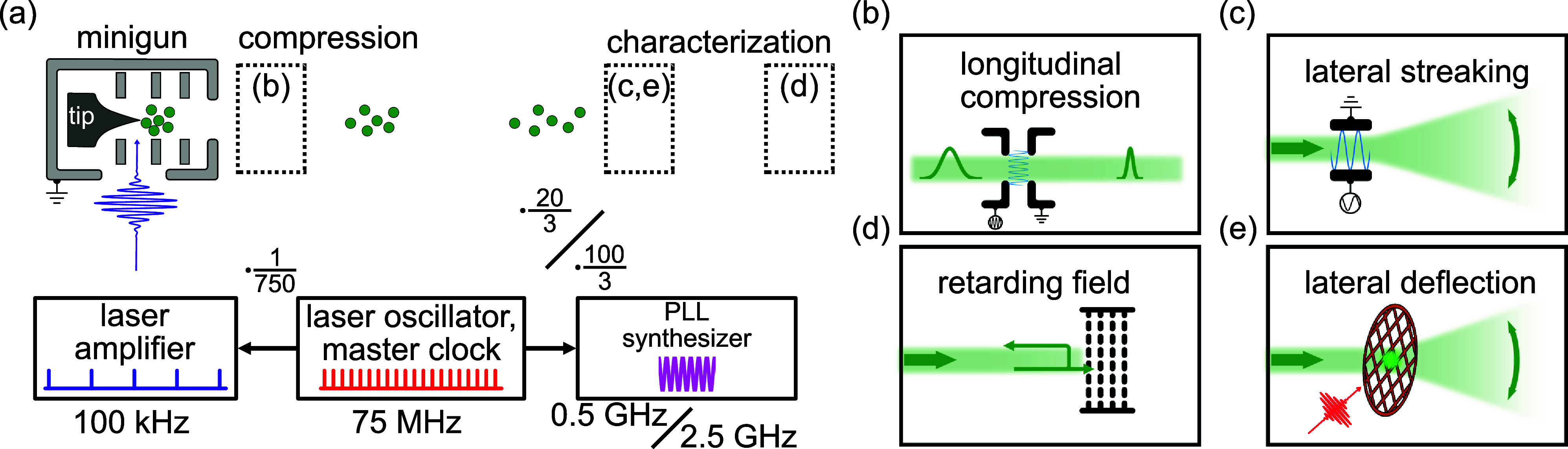
Experimental setup with four different control and characterization configurations. (a) Schematic illustration with electron gun, electron propagation, employed frequencies and laser synchronization, as well as dashed boxes, which represent locations of elements placed for different measurements. Sketches of (b) the longitudinal compression, (c) the lateral streaking, (d) the retarding field analyzer, and (e) the lateral deflection.

In the experiments, we combine a millimeter-sized electron gun^11^ with a pulse-compression cavity (Fig. 6 in the Appendix). Electron pulses are stroboscopically generated by photoemission from a tip-shaped photocathode.^74,75^ The electron pulses are accelerated and collimated by an electrostatic lens system.^11,76^ Further typical beam parameters are summarized in Table II in the Appendix. During propagation in free space, the initial energy spread within the pulses, further enhanced by electron–electron interactions, leads to dispersion (pulse chirp) and an increase in the electron pulse duration, see Fig. 2(a). The spatial electron-beam profile is measured using a microchannel plate (MCP) and phosphor-screen detector assembly.

**TABLE I.** Average energy and broadening for selected RF phases leading to extrema in energy or broadening.

Description	Mean kin. energy $\bar{E}$/eV	Energy width ΔE/eV
Uncompressed	81	1.0
Acceleration	94	2.0
Deceleration	71	3.0
Compression	83	11.4
Stretching	82	13.0

**FIG. 3.**
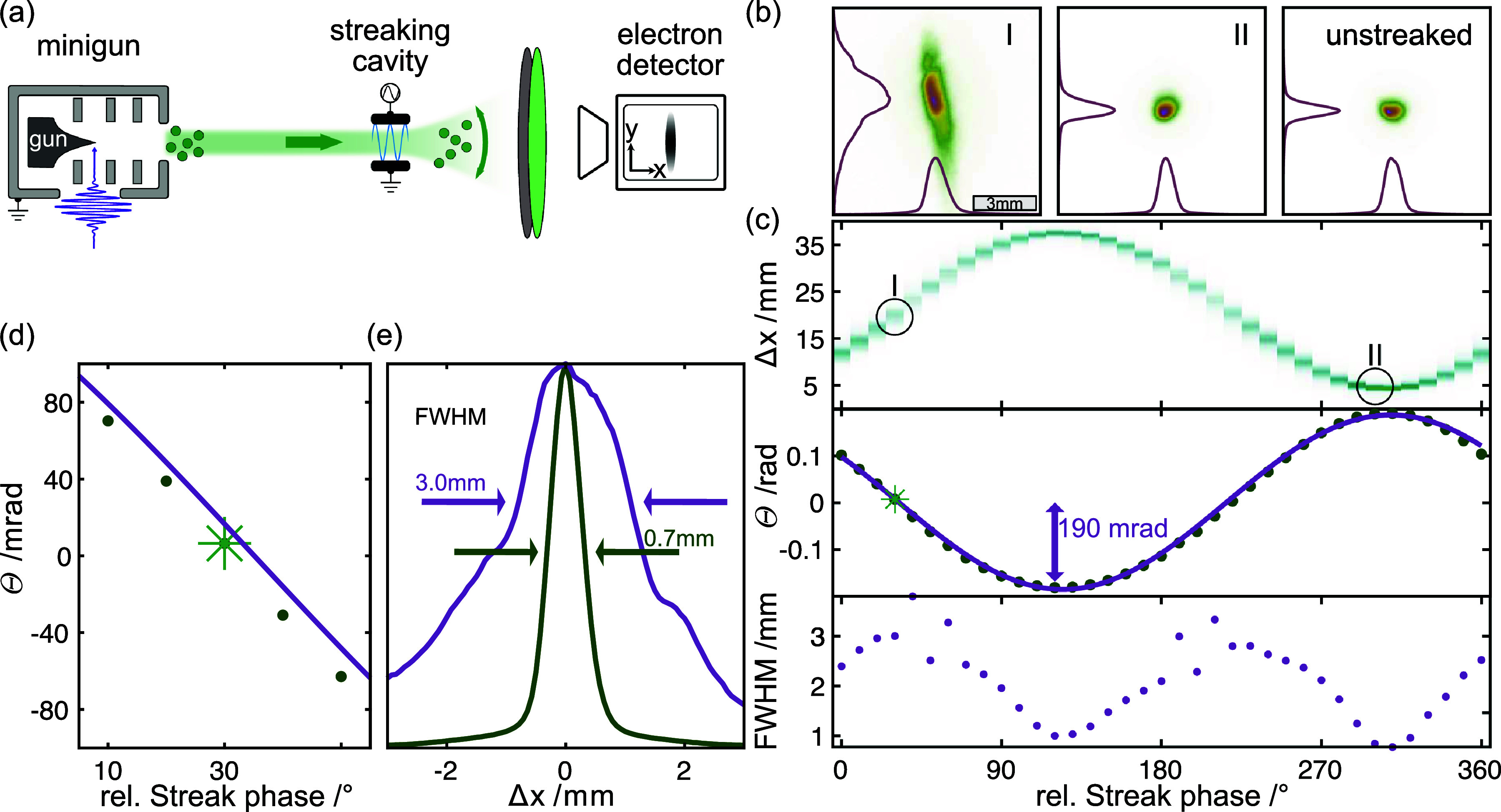
Radio frequency streaking of low-energy electron pulses. (a) Schematic setup with electron gun, streaking cavity and detector. (b) Streaked electron beam profile (I: maximally broadened; II: maximally deflected). Unstreaked image shown for comparison. (c) Electron beam profile (top), evaluated deflection (center; solid line is a sinusoidal fit) and broadening (bottom) as a function of the streaking field phase. (d) Magnified view of the region of negative slope in deflection angle; the star marks the phase with the maximum broadening (b-I). (e) Beam profiles along the streak direction for the phases of maximum (blue) and minimum (green) broadening.

Four different schemes are used to characterize and manipulate the electron pulse, namely, longitudinal compression, lateral streaking, retarding-field spectroscopy, and deflection by laser-induced transient electric fields (TEFE),^77^ as illustrated in Figs. 2(b)–2(e). A streak cavity [Fig. 2(c)] is placed in the beam path to demonstrate the laser-to-RF phase stability and to obtain a first characterization of the electron pulse duration. This cavity converts the electron arrival time into a laterally streaked distribution, which is detected by the MCP. The compression cavity, on the other hand, shapes the electron energy distribution of the electron pulses in the longitudinal direction. The applied time-dependent electric field in the compression cavity manipulates the longitudinal phase-space distribution, leading to a reversal of the electron-pulse chirp and resulting in a temporal focus upon further propagation [cf. Fig. 2(b)]. For both cavities, the synchronization between the electron pulses and the electric fields is of central importance. In the present study, the laser oscillator acts as a master clock for a phase-locked loop (PLL) synthesizer, which generates phase-stable sinusoidal signals for compression and streaking with adapted frequencies.

Characteristic pulse properties, namely, the mean energy ${\bar{E}}_{\text{kin}}$ and the energy width ΔE [Fig. 2(d)] are quantified with a self-built retarding-field analyzer. This allows us to measure energy spectra of an uncompressed pulse and after action by the compression cavity. Figure 2(e) illustrates a lateral deflection induced by TEFE in a pump-probe setup to measure the temporal resolution of the compressed pulses.

## III. LOW-ENERGY ELECTRON STREAKING

In a first set of experiments, sketched in Fig. 3(a), we employ transverse streaking in the absence of a compression cavity, to demonstrate successful laser-to-RF synchronization, and to characterize the uncompressed electron pulse duration. The following results are obtained with 100-eV electron pulses (rep. rate of 100  kHz and 1–2 electrons per pulse^11^), which are passed through the streak cavity, undergo a deflection, and are then detected after a drift distance of about 7 cm.

**FIG. 4.**
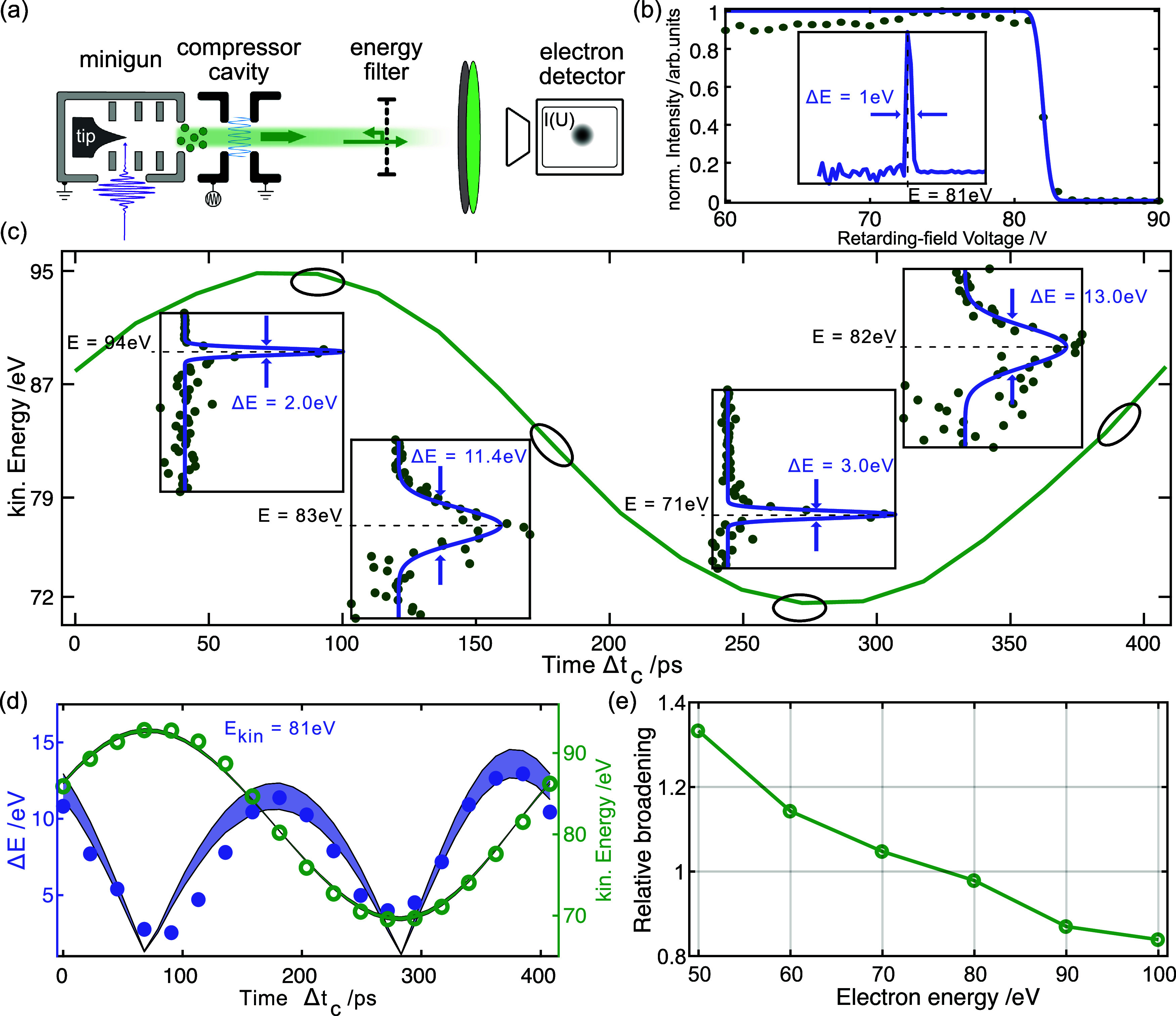
Retarding-field analysis for measuring the mean kinetic energy ${\bar{E}}_{\text{kin}}$ and the energy width ΔE of the electron pulses. (a) Schematic setup with electron gun, compression cavity, energy filter, and detector. (b) Normalized intensity plotted against the retarding field voltage for an uncompressed pulse, with the derivative yielding the electron kinetic energy spectrum. (c) Measured kinetic energy for different compression cavity times; insets show four different extreme cases, drastic change in ${\bar{E}}_{\text{kin}}$ (first and third) or in ΔE (second and fourth). (d) ΔE and ${\bar{E}}_{\text{kin}}$ as a function of the RF time. The colored area illustrates the effect of initial pulse durations (55−65 ps) when entering the compression field for kinetic energy and width. (e) Relative broadening for different electron energies.

The cavity is excited by a continuous-wave electric field at a frequency $f_{\text{str}}=0.5\;\text{GHz}$, generated by a synchronized PLL-synthesizer. The phase at which an electron enters the streak cycle is translated into the angular deflection it experiences. For an electron ensemble composed of a train of one- to few-electron pulses, the sinusoidal phase-dependence is convoluted with the arrival-time distribution. As a result, at the phases of maximum angular deflection, the electron ensemble experiences a deflection that only weakly depends on time, such that the beam shape is narrow, and is almost unchanged [compare Figs. 3(b-II) and 3(b) unstreaked]. In contrast, for the phases with the fastest change in deflection, namely, the zero-crossings, the beam is maximally streaked, and the arrival-time distribution (i.e., electron pulse duration) is mapped onto the transverse momentum. This results in a significant spatial beam broadening on the detector [Fig. 3(b-I)].

Figure 3(c) (top) shows the phase-dependent final beam profile along the streak direction, highlighting the phases of maximum streaking (I) and deflection (II). Each image is integrated over 12.5 s, i.e., $1.25\times 10^{6}$ laser pulses at the repetition rate of 100  kHz. The phase-dependent deflection angle and the full-width-at-half-maximum (FWHM) of the beam along the streak direction are shown in the panels of Fig. 3(c) (center) and (bottom), respectively. Figure 3(d) magnifies the positive slope of the angular deflection with a sinusoidal fit (solid line) and indicates the streak phase with the maximum broadening (green star). The measured FWHM of the beam profile along the streak direction [Fig. 3(e)] differs significantly for an unstreaked ( $x_{0}=0.7\;\text{mm}$) and a maximally streaked beam ( x=3.0 mm). The amplitude of the angular deflection trace and the streak frequency yield a streak rate $R_{x}=53\;\mu m/\text{ps}$, from which we estimate the pulse duration via $\tau_{\text{str}}\approx 1/R_{x}{\left(\Delta x^{2}-x_{0}^{2}\right)}^{1/2}$. We obtain a pulse duration of $\tau_{\text{str}}=55\;\text{ps}$ for a propagation distance of 10 mm. (For an average bunch charge on the order of only one electron, the pulse duration denotes the width of an arrival time histogram with respect to the reference time given by the driving laser pulse.) This value is somewhat larger than the minimal pulse durations obtained with a nominally identical gun geometry,^11^ which is likely caused by using a blunter tip in this case (radius r=190 nm measured by scanning electron microscopy).

## IV. LONGITUDINAL PHASE SPACE CONTROL OF LOW-ENERGY ELECTRON PULSES

In this section, we discuss the RF-based manipulation of the electron kinetic energy spectrum, which will be essential for temporal electron-pulse compression. Specifically, we characterize the average kinetic energy ${\bar{E}}_{\text{kin}}$ and the spectral broadening ΔE induced by a compression cavity as a function of the RF-phase. We measure these parameters by placing a retarding-field energy analyzer in the beam path, as sketched in Fig. 3(a). In this device, a variable voltage is applied to a grid between two ground-potential grids. The transmitted electron current Φ is recorded upon scanning the voltage *U*, and electron spectra are obtained by a numerical derivative.^78–80^ For simplicity, we extract the average electron energy and the broadening by fits of the measured voltage scans to error functions (see the Appendix). The compression cavity is excited by an alternating field at a frequency $f_{\text{comp}}=2.5\;\text{GHz}$. The arrival times $\Delta t_{c}$ of the electrons in the compression-cycle affect the average energy and the broadening of the distribution. As a reference, the retarding-field measurements are performed for the uncompressed electron pulses. Figure 4(b) shows both the measured voltage-dependent intensity trace and the corresponding derivative of the data points, which yield ${\bar{E}}_{\text{kin}}=81$ and ΔE=1.0 eV.

**FIG. 5.**
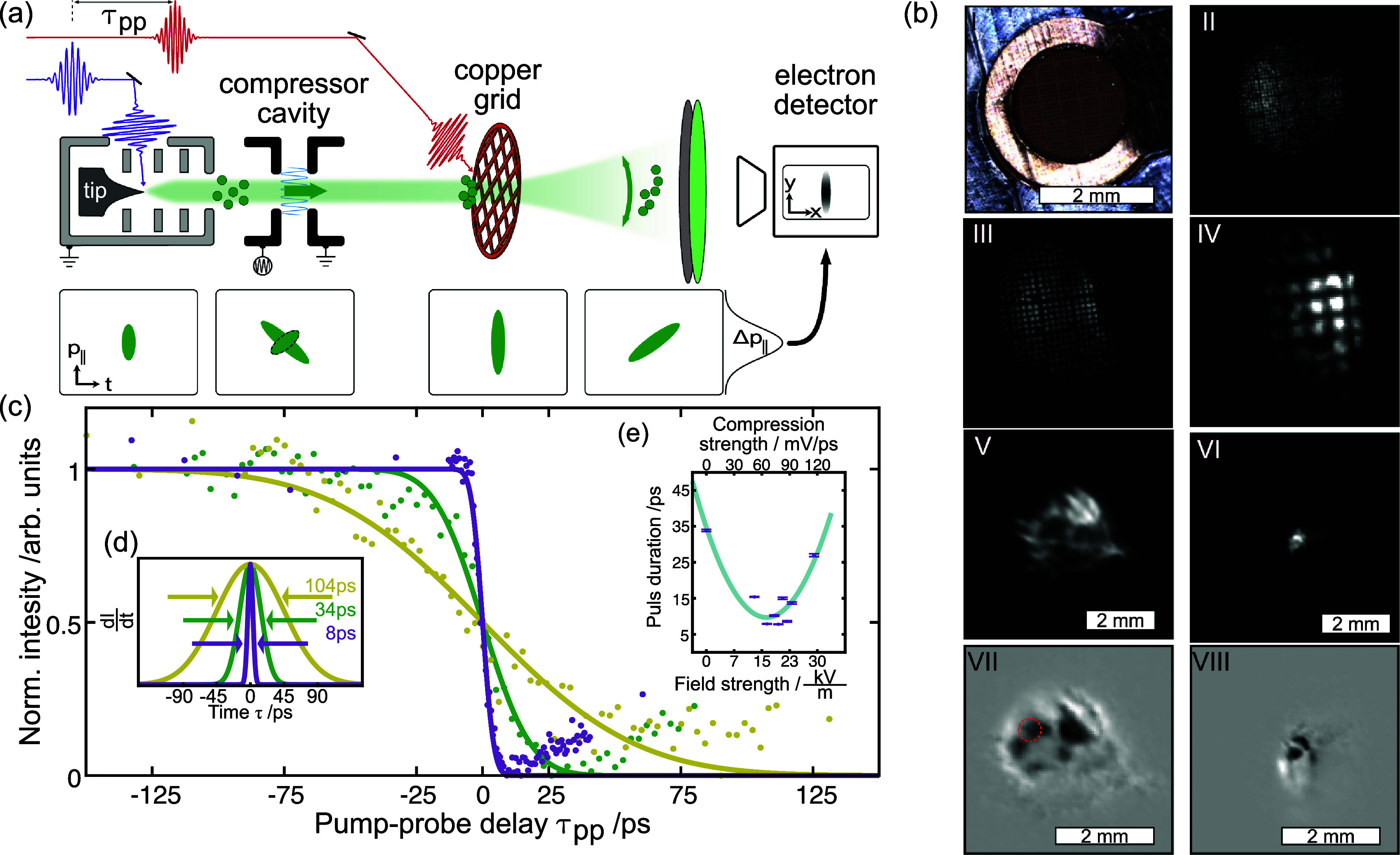
Measurement of electron pulse duration and compression using transient electric fields. (a) Schematic of the setup with electron gun, compression cavity, metal grid, and detector. Sketch of four phase-space distributions throughout the propagation. (b) Photograph of the metal grid; electron projection images of the grid, varying the beam divergence with the gun lens and the grid-gun distance for different magnifications (II)–(VI); with a nearly collimated beam, the transmission image becomes less regular (V); and no grid features are seen for the collimated beam (VI). Images (VII) and (VIII): change of image intensity compared with images (V) and (VI), respectively, induced by a pump-laser-generated electron cloud that distorts the transmitted beam by the transient electric field effect (dark area: intensity decrease, bright area: intensity increase). A delay-dependent multimedia file of a movie is available online. Dashed circle: analysis area of $0.38\;{\text{mm}}^{2}$ exhibiting rapid changes used for pulse characterization in (c). All scale bars refer to the size on the detector after a 7  cm drift distance. (c) Pump-probe delay traces showing the normalized intensity in the analysis area for uncompressed (green), actively stretched (yellow), and compressed (purple) pulses. (d) Derivative of fit curves from (c). (e) Retrieved pulse duration for varying compression field strengths. Multimedia available online.
10.1063/4.0000235.1

Measured for different RF times (in time steps of 23 ps), we find a sinusoidal variation of the kinetic energy. As the spectral broadening scales with the derivative of the field, its variation approximates the absolute value of a cosine function [Fig. 4(d)]. Figure 4(c) shows the distributions at four characteristic values of the RF-phase, for which extrema in the energy or width are obtained (Table I). These phase-dependent changes of the electron energy distribution are, as expected, analogous to that of the transverse modulation in the streak experiment. In analogy to the case of transverse streaking, we estimate an initial pulse broadening based on the streak rate $R_{E}=5.7\;\;\text{ps}/\text{eV}$ (see the Appendix for details). We obtain a pulse duration of 65  ps, consistent with the estimate from transverse streaking.

**TABLE II.** Summary of typical electron beam parameters.

Beam parameter	Value
Pulse charge	≈2 e/pulse^11^
Electron energy	50–120 eV
Repetition rate	100 kHz
Kinetic energy width	1 eV
Pulse duration:	at E=80 eV, distance: 8 mm
Uncompressed	34 ps
compressed	8 ps
Beam diameter:	
Uncompressed (on MCP)	<600 *μ*m [Fig. 9(a)]
Uncompressed (on sample)^a^	<100 *μ*m^11^
Compressed	1.3 mm [Fig. 4(b-VI)]

^a^ Obtained with the same electrostatic design but a different individual gun and tip. Measured by pump-probe overlap.^11^

Notably, we find a difference between the two extrema in broadening at the fastest change in average energy [cf. Fig. 4(d)], which is a result of the pre-existing chirp of the injected pulse. The phase of temporal compression partially compensates this chirp and thus leads to a slightly narrower final spectrum. In addition, Fig. 4(d) shows the results of particle trajectory simulations for the average energy and the broadening (lines and shaded areas). The computations reproduce the experimental findings, and the boundaries of the shaded areas correspond to the energies and energy differences obtained for electrons separated in time by the pulse durations estimated from the streak ( 55 ps) and retarding-field ( 65 ps) experiments (see the Appendix for details).

To demonstrate the applicability of the compression setup in the context of an ULEED experiment, these measurements were carried out across an energy range from 50 to 100 eV with the same cavity settings. The data (Appendix, Fig. 13) show that the amplitude $A_{\text{eff}}$ of the kinetic energy modulation increases with increasing electron energy, whereas the energy width decreases in the corresponding compression phase. This also leads to a decrease in the relative broadening $\Delta E/A_{\text{eff}}$ with increasing electron energy [Fig. 4(e)]. The primary cause of this observation is the reduced pulse dispersion for higher electron energies. In order to reduce the injection pulse duration for optimized compression, for the following experiments, the tip was replaced by one with a sharper radius of curvature ( r=27 nm).

## V. LOW-ENERGY PULSE COMPRESSION ANALYZED BY TRANSIENT ELECTRIC FIELDS

In order to quantify the impact on the electron pulse duration by the compression cavity, the experimental setup is modified as shown in Fig. 5(a) (Multimedia view). Here, a copper grid [Fig. 5(b-I)] is placed in the beam path at a distance of about 10 mm from the electron gun. We initially defocus the electron beam to project an image of the grid onto the detector. A sequence of the projection images at varying lens voltage and corresponding magnifications can be seen in Figs. 5(b-II)–5(b-IV). For the time-resolved measurements, the lens voltage is adjusted close to beam collimation, and the distance to the grid was optimized [Fig. 5(b-V)] for the shortest pulse duration. An optical pump pulse focused on the grid produces an electron space-charge cloud that distorts the projection image [Fig. 5(b-V)]. By varying the time delay $\tau_{\text{pp}}$ between the laser pump and electron probe pulses, the generation and expansion of the electron cloud can be tracked. To characterize the pulse duration, the beam intensity in a region with rapidly varying contrast [dashed circle in Figs. 5(b-V)–5(b-VI)] is plotted against $\tau_{\text{pp}}$. Changes in intensity are fitted with an error function. In this way, we first characterize the duration of an uncompressed pulse [Fig. 5(c), green curve], which yields τ=34 ps. Next, in the application of the compression cavity, we repeat the measurement at phases of maximum temporal compression [Fig. 5(c), purple] and stretching [Fig. 5(c), yellow]. At the phase of temporal stretching, the electron pulse duration increases threefold, reaching $\tau_{\text{stretched}}=104\;\text{ps}$. Optimizing the phase for maximum compression, we find a fourfold decrease down to $\tau_{\text{compressed}}=8\;\text{ps}$. The corresponding Gaussian derivatives of the three error function fits are displayed in Fig. 5(d).

**FIG. 6.**
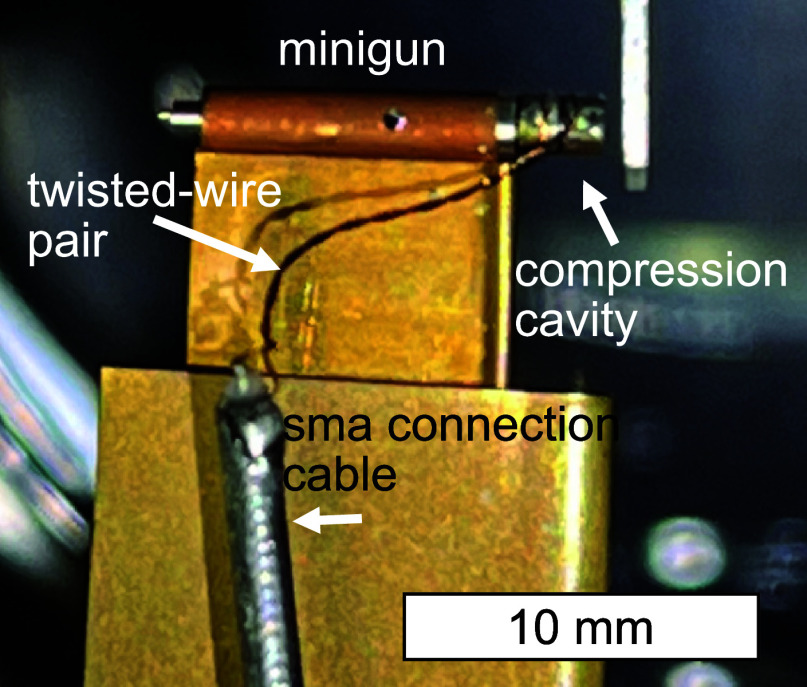
Minigun with integrated compression cavity, twisted-wire pair, and SMA connection cables.

Figure 5(e) displays the measured electron pulse duration as a function of the cavity field strength for 80 eV electrons and a fixed grid location. The location of the temporal focus is controlled by the applied field strength, and the strongest compression is obtained for a cavity field strength of 28 kV/m.

We performed this pulse characterization for additional electron energies of 100 and 120 eV without changing the grid position. The previous experiments illustrated that a change in electron energy has a significant effect on compression efficiency. Therefore, the compression field strength was adjusted ( 24 and 30 kV/m) to shift the temporal focus to the grid position. Minimal pulse durations of $\tau_{100\;\text{eV}}=11$ and $\tau_{120\;\text{eV}}=12\;\text{ps}$ were measured for the given distance. Although large amplitudes of the compression signal should result in an optimized pulse duration, we encountered charging effects limiting the maximum usable field amplitudes. It should be noted that shorter pulses durations, down to 1 ps,^53^ have previously been achieved for microfabricated electron guns. While the scheme can also be employed for such guns, the present approach provides intrinsic advantages in terms of a mechanically very robust and monolithic design.

Presently, the minimal pulse durations are limited by various contributing factors, including the injection pulse durations of the millimeter-sized photoelectron gun, electronic phase jitter of the used electronics, and intrinsic beam properties as well as spatial variations of the compression fields. A dominant influence is expected from electron energy variations due to longitudinal beam emittance and spatially inhomogeneous compression fields across the beam profile (see also Subsection 7 of the Appendix).

## VI. SUMMARY AND OUTLOOK

In conclusion, we presented the construction and first demonstration of pulse compression at low electron kinetic energies. Several characterization and control methods of the transverse and longitudinal phase-space distribution were employed. Using a self-built RF cavity, a fourfold compression to a minimum pulse duration of τ=8 ps is achieved for 80 eV electron energy. In order to employ the scheme for various electron energies in a single geometry, adapting the compression amplitude, frequency, and phase to the electron energy will be desirable. Transferring the compression scheme to micrometer-scale electron guns and reducing electronic jitter, we anticipate that temporal resolutions in ultrafast low-energy electron diffraction deep in the femtosecond regime can be reached.

## Figures and Tables

**FIG. 7.**
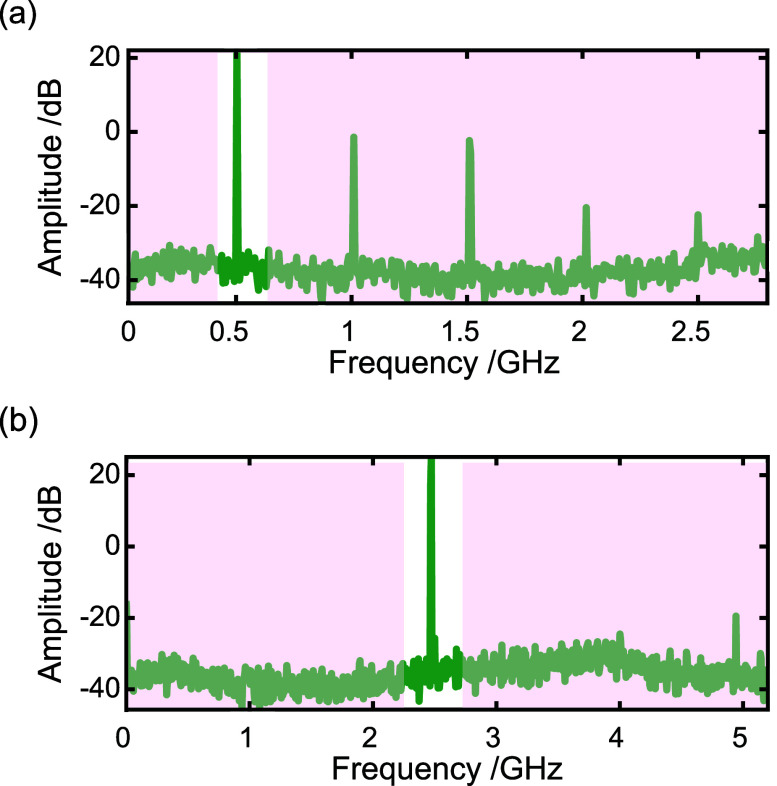
(a) PLL synthesizers generated 0.5 GHz spectra for the streak cavity. (b) Spectra of the 2.5 GHz compression cavity signal.

**FIG. 8.**
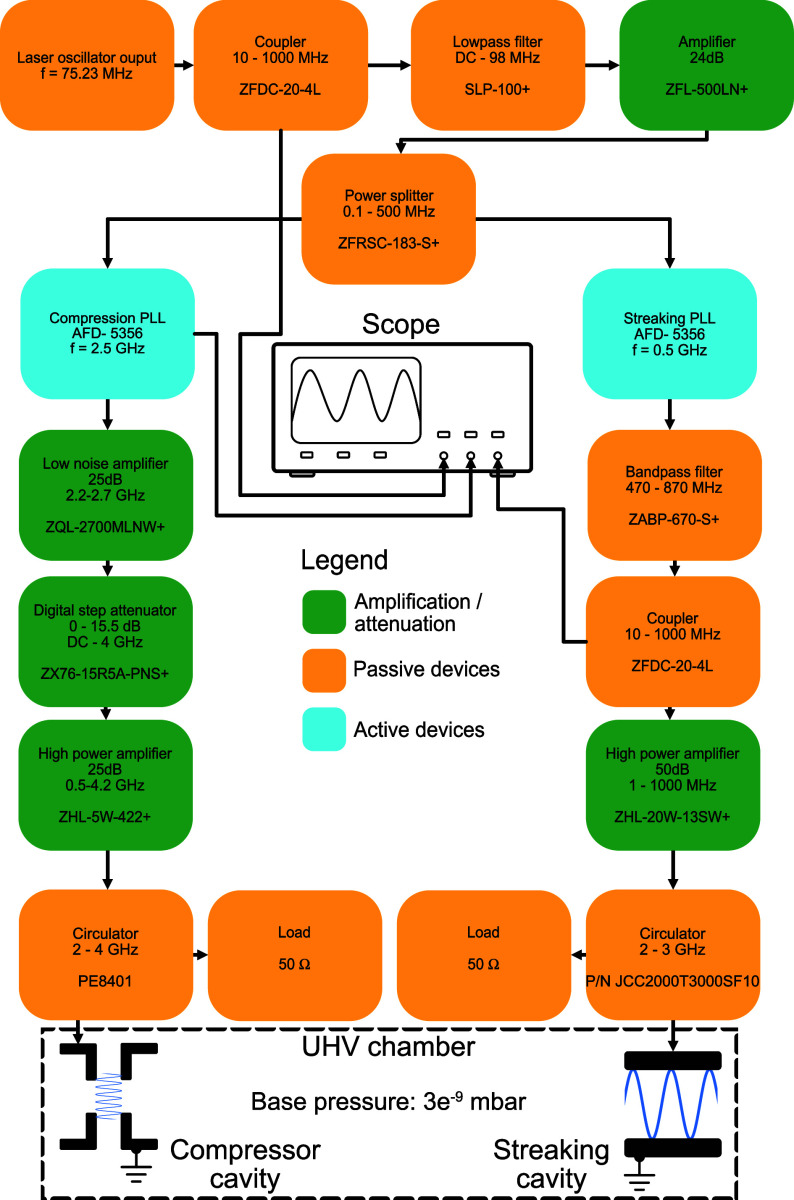
Electronic synchronization system with compression- and streak-cavity. The different colors represent active/passive devices and amplifiers/attenuators.

**FIG. 9.**
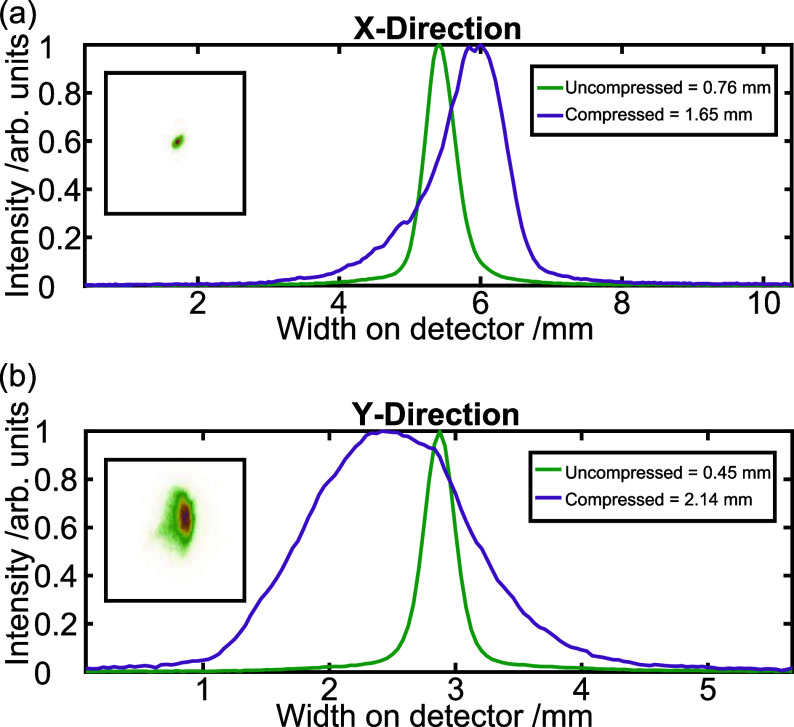
(a) Beam spot profile in horizontal direction on the detector (inset: uncompressed beam on detector). (b) Beam-spot profile in vertical direction (inset: compressed beam on detector). The lens settings are unchanged. Some of the aberrations shown are a result of defocusing, which can be corrected by adjusting the lens voltages [Fig. 4(b-VI)]

**FIG. 10.**
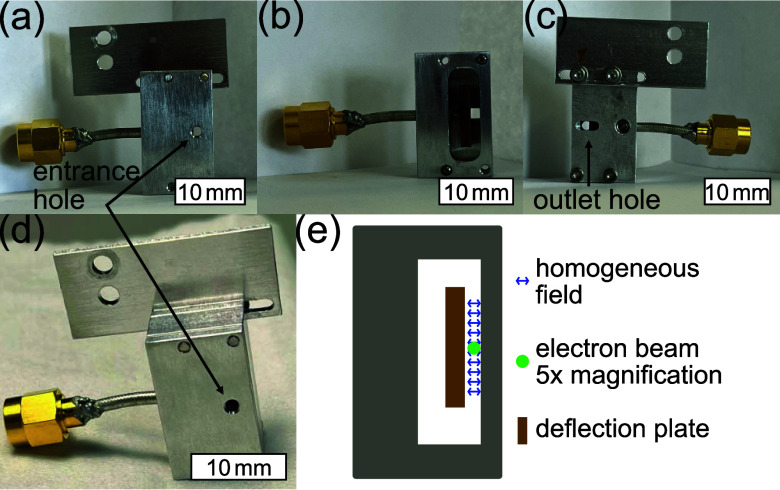
(a) Streak cavity housing facing electron source (circular hole). (b) The PLL signal is connected directly to the deflection plate via an SMA cable. (c) Cavity housing facing the MCP (oval hole). The oblong hole in the shielding plate is to prevent cutting the maximum deflected electrons. (d) The assembled streak cavity from the electron beam direction. (e) Sketch of (b) with homogeneous field (blue arrows), fivefold magnification of the electron beam (green circle), and the deflection plate (brown rectangle).

**FIG. 11.**
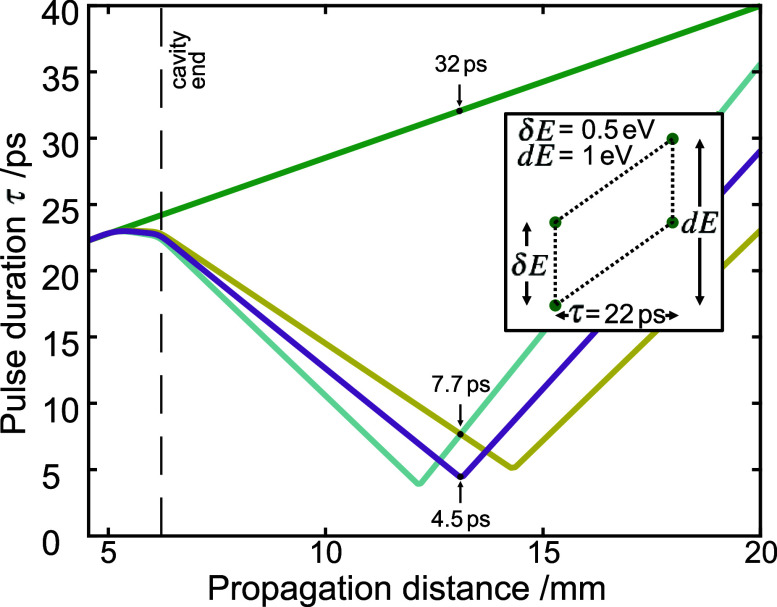
Calculated pulse duration during propagation for different compression cavity amplitudes. In this simple model, four electron trajectories are considered. In the absence of a compression field (green), we find a pulse duration of 32  ps after a 7  mm drift distance. Evolution of pulse duration in the presence of compression (purple), and upon variation of the magnitude of the compression field by ±12% (cyan and yellow). The inset illustrates the injection pulse condition used for the computation.

**FIG. 12.**
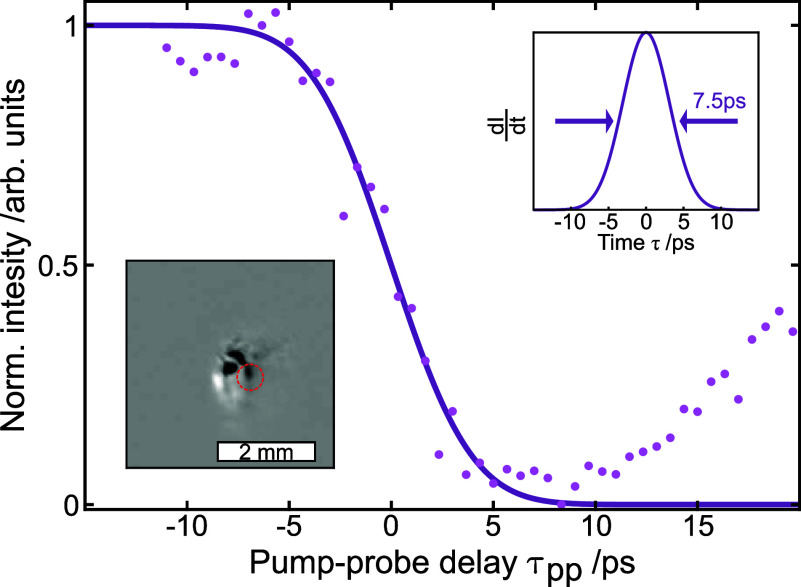
Pump-probe delay traces showing the normalized intensity in the analysis region for a compressed pulse for a collimated electron pulse. The insets show the derivative of the fitted curve and an electron projection image (cycle: analysis area of $0.38\;\;{\text{mm}}^{2}$).

**FIG. 13.**
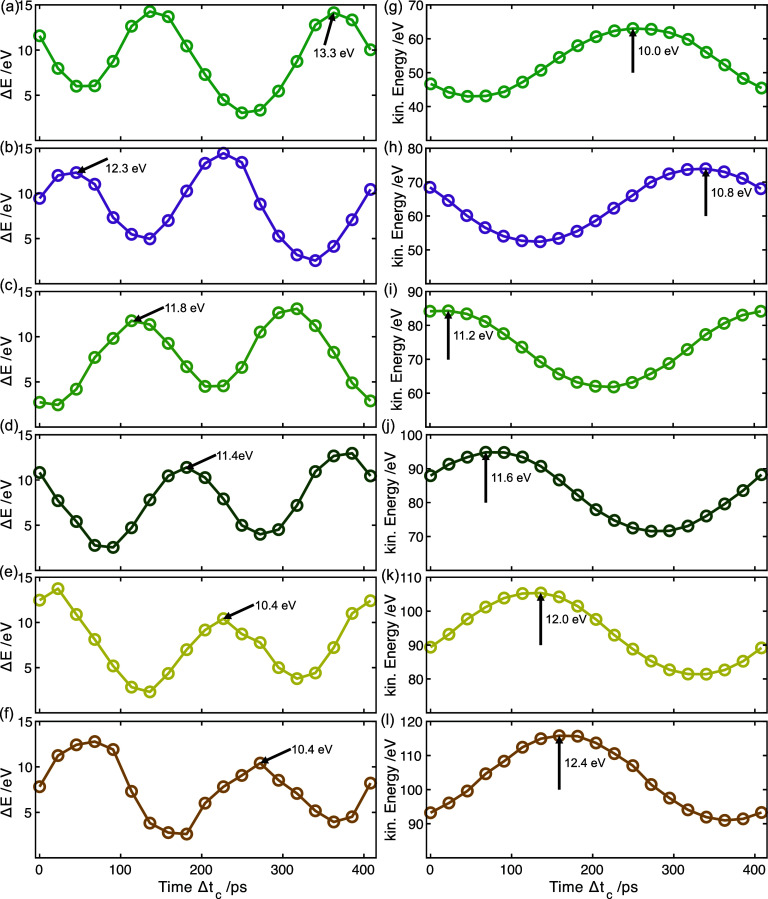
(a)–(f) Measured energy width ΔE for (a) 50, (b) 60, (c) 70, (d) 80, (e) 90, and (f) 100 eV. A shift in the two maxima directly corresponds with the velocity change of the electron pulses. This shift is also visible in the kin. energy ${\bar{E}}_{\text{kin}}$ data.

## Data Availability

The data that support the findings of this study are available from the corresponding author.

## APPENDIX: METHODS

### 1. Laser system

The optical setup [see Fig. 2(a)] provides a synchronization signal as well as pump pulses ( $\lambda_{c}=1030\;\text{nm}$, repetition rate $f_{\text{rep}}=100\;\text{kHz}$, average power $P_{1030nm}=36\;\text{mW}$, pulse duration Δτ=212 fs) for the grating excitation. Photoelectron pulses are produced using the second harmonic of the signal of a non-collinear optical parametric amplifier (NOPA, tunable between λ=325−450 nm, pulse duration Δτ=15−50 fs).

### 2. Electron emission

High-coherence electron pulses are emitted from a nanometric tungsten tip photocathode. The frequency-doubled NOPA output at a wavelength of λ=400 nm (average power: 500 μW, 100 kHz rep. rate) is focused by a plano–convex lens (focal length l=300 mm) onto the tip and lead to the emission of about 1–2 electrons per laser pulse.^11^ In a suppressor–extractor geometry, the electrons are accelerated and collimated by an additional Einzel lens [see Fig. 2(a) and 6]. For the analyzed measurement data, most images were integrated over 8– 12.5  s, which corresponds to millions of laser pulses at the repetition rate of 100  kHz; see additional beam parameters in Table II.

### 3. Radio frequency field generation and synchronization for electron pulse compression and streaking

The phase stability between the emitted electrons and the time-dependent electric field of the compression cavity is crucial for active pulse compression. A phase-locked loop synthesizer (output frequency f=53.125 MHz– 13.6 GHz) provides such phase stability [see Fig. 2(a)]. A built-in voltage-controlled oscillator generates the required frequency of $f_{\text{streaking}}=0.5\;\text{GHz}$ or $f_{\text{compression}}=2.5\;\text{GHz}$ for streaking and compression experiments, respectively. For phase synchronization, a photodiode output signal from the laser oscillator at a frequency of $f_{\text{laser}}=75\;\text{MHz}$ is used as input and reference signal (compare with Fig. 2). Figure 7 provides an overview of the used electronic parts. In a first step, the photodiode signal is cleaned with a filter and amplified to supply signals to both PLLs. Figures 7(a) and 7(b) show the corresponding spectra of the generated sinusoidal signal. The shadowed area indicates the frequency range suppressed by bandpass filters and selective amplification. Depending on the output frequency, different post-processing requirements are needed, including amplifier stages or bandpass filters. A detailed sketch is given in Fig. 8. In terms of phase and amplitude jitter, the dominant source comes from electronics and laser-RF synchronization. The electronic phase jitter was quantified using a 16-GHz oscilloscope, yielding a value of about 2  ps. Compared to this, amplitude jitter represents a negligible contribution.

### 4. Compression cavity

The custom compression cavity consists of two copper electrodes separated by a 130 μm thick insulating spacer. The dimensions of these electrodes are the same as the minigun electrodes, diameter d=2 mm and length l=1.2 mm [see Fig. 4(b)], which preserves the outer diameter of the electron source. The compression signal is supplied by a flexible twisted-wire pair cable, which minimizes vibration transmission to the minigun. A reduction of efficiency losses due to standing waves or impedance mismatches is ensured by a cable length ( 2 cm) well below the wavelength of the RF signal.

Figure 9 shows a compression-induced deformation of the electron beam profile, measured using the setup shown in Fig. 4. We observe a slightly asymmetric deflection and distortion, most prominently in the vertical direction. With a broadening by a factor of about 2 and 4 in the horizontal and vertical direction, respectively, the beam quality remains sufficient to carry out time-resolved diffraction experiments in the future. Moreover, some of the broadening is due to a defocusing, which we found can be corrected by modifications of the lens settings [cf. Fig. 5(b-VI)].

### 5. Streaking cavity

A flat copper electrode with a distance of d=2 mm to the housing and a width of w=3 mm [see Fig. 10(b)] is placed in the electron beam path. The copper plate is attached directly to the central conductor of an SMA cable. The outer conductor is connected to the housing via a screw [see Fig. 10(c)]. The alternating field with a frequency of $f_{\text{streak}}=0.5\;\text{GHz}$ is generated between the plate and the housing. The intracavity field strength can be estimated from the observed displacement angle, resulting in a deflection-field amplitude of E=8 V/mm. The corresponding voltage applied to a plate capacitor is 32  V peak-to-peak.

The associated transverse streak rate is

$$R_{x}=\frac{1}{2\pi \;f_{\text{streak}}\;A_{\text{deflection}}},$$
(A1)

with the deflection amplitude $A_{\text{deflection}}=17\;\text{mm}$.

### 6. Retarding-field analyzer

During the measurement, a retarding field suppresses the transmission of electrons with energies below the mean kinetic energy. Increasing the voltage of the retarding field *U* leads to a drop in the detected intensity Φ. This behavior can be well described by an error function,

$$\Phi (U)=\frac{1}{2}\left(1+\text{erf}\left(\frac{2\;\sqrt{\;\text{log}(2)}\left(U-{\bar{E}}_{\text{kin}}\right)}{\Delta E}\right)\right).$$
(A2)

Here, the electron pulse's average kinetic energy is defined by the retarding voltage at 50% of the maximum intensity, and the energy width ( ΔE) is determined by the error function width.

The phase-dependent change in kinetic energy is approximated by

$$\bar{E}=A\;\;\text{sin}\left(2\pi f_{c}\;t_{c}\right).$$
(A3)

At the zero-crossing of the field, the rate of energy change is $R_{E}=1/(2\pi \;f_{c}\;A)$ (analogous to the streak rate $R_{x}$), and we estimate the pulse duration of electrons entering the compression cavity to τ=65 ps, via $\tau \approx dE\;R_{E}$, with the measured amplitude A=11.4 eV [Fig. 4(c)], the frequency $f_{c}=2.45\;\text{GHz}$, and the experimentally determined spectral width in the compression phase d E=11.4 eV [Fig. 4(c) second inset].

Particle trajectory simulations using the experimental geometry are performed using an ODE solver. The electric field along the beam axis is calculated by a finite element simulation. The kinetic energy of each electron is determined as a function of its entrance phase into the compression field. The measured electron-beam parameters are used as input, namely, the initial energy $E_{0}=81\;\text{eV}$, the energy width ΔE=1 eV, and the time separation τ=±30 ps. For simplicity, we compute four characteristic electron trajectories with differential changes in the initial time and energy. The resulting kinetic energy distribution is described in terms of its average and maximum energy separation $\Delta E=\text{abs}(E_{\text{kin},\text{max}}-E_{\text{kin},\text{min}})$ for each RF phase.

### 7. Limitations in minimal pulse duration

Given the high electron dispersion [ 2.4  ps/(mm eV) at 80-eV energy], variations in electron kinetic energy are likely to be a dominant factor in the minimum achievable pulse duration. These can be divided into intrinsic beam properties and spatial variations of the compression field across the beam profile.

One contributing factor will be imperfect longitudinal electron phase space distribution at the position of the compressor, or increased longitudinal beam emittance. Specifically, due to a variety of influences, which include the emission process and off-axis trajectories, at any position along the dispersed electron pulse, there will be a finite energy width *δE*. Remaining dispersion according to this energy width will limit the compressed pulse duration even under otherwise ideal conditions. For a momentary energy width along the dispersed pulse of dE=1  eV, we computed a minimum achievable pulse duration of about 4.5  ps for the given geometry and compression field (see Fig. 11, purple line). As in the other simulations, for simplicity, we used four characteristic trajectories and plot their maximum temporal separation along the beam path.

Next, spatially inhomogeneous compression fields represent a contribution to the overall pulse duration. Specifically, the energetic broadening at the extrema of the average kinetic energy provides us with an estimate on the variation of field strengths experienced by different trajectories in the compression unit. For an 11-eV amplitude of the kinetic energy variation and an energy broadening of about 1  eV in the absence of compression, we find a broadening to 2 and 3  eV at maximum acceleration and deceleration, respectively. The kinetic energy spectra measured are slightly asymmetric toward the center energy, which imply that at least some of this broadening stems from the finite injection pulse width (different electrons experiencing different phases). However, a spatial variation of the compression field across the beam will also contribute some broadening. In order to estimate this influence, we computed trajectories for a variation of the compression field by ±12%. This increases the minimal pulse duration to 7.7  ps. There is good agreement with the experiment in both the uncompressed and compressed calculations.

### 8. Transient electric field effect

A copper transmission electron microscope (TEM) grid, with a bar thickness of 20 μm, aperture size of 43 μm, and pitch of 63 μm, is placed in the beam path at a variable distance from the minigun. In a stroboscopic optical-pump, electron-probe scheme with a variable time delay $\tau_{\text{pp}}$, the grid is excited by a laser pulse (center wavelength $\lambda_{\text{pump}}=1030\;\text{nm}$) with a pulse fluence of $2\;\text{mJ}/{\text{cm}}^{2}$, causing a rapidly evolving electron cloud. The resulting distortion of the projection image is recorded and can be as fast as 1 ps.^26,36,77,81^

An additional pump-probe delay trace is shown in Fig. 12, where the fastest feature exhibits a similar time constant as in Sec. V.
